# Elbasvir/grazoprevir for hepatitis C virus genotype 1b East-Asian patients receiving hemodialysis

**DOI:** 10.1038/s41598-020-66182-8

**Published:** 2020-06-08

**Authors:** Chen-Hua Liu, Cheng-Yuan Peng, Yu-Jen Fang, Wei-Yu Kao, Sheng-Shun Yang, Cheng-Kuan Lin, Hsueh-Chou Lai, Wen-Pang Su, Sheng-Uei Fang, Chun-Chao Chang, Tung-Hung Su, Chun-Jen Liu, Pei-Jer Chen, Ding-Shinn Chen, Jia-Horng Kao

**Affiliations:** 1 Division of Gastroenterology and Hepatology, Department of Internal Medicine, National Taiwan University Hospital, Taipei, Taiwan; 2 Hepatitis Research Center, National Taiwan University Hospital, Taipei, Taiwan; 3 Division of Gastroenterology and Hepatology, Department of Internal Medicine, National Taiwan University Hospital, Yun-Lin Branch Douliou, Taiwan; 4 School of Medicine, China Medical University, Taichung, Taiwan; 5 Center for Digestive Medicine, Department of Internal Medicine, China Medical University Hospital, Taichung, Taiwan; 6 Division of Gastroenterology and Hepatology, Department of Internal Medicine, Taipei Medical University Hospital, Taipei, Taiwan; 7 Division of Gastroenterology and Hepatology, Department of Internal Medicine, School of Medicine, College of Medicine, Taipei Medical University, Taipei, Taiwan; 8 Graduate Institute of Clinical Medicine, College of Medicine, Taipei Medical University, Taipei, Taiwan; 9 Division of Gastroenterology and Hepatology, Department of Internal Medicine, Taichung Veterans General Hospital, Taichung, Taiwan; 10 School of Medicine, Chung Shan Medical University, Taichung, Taiwan; 11 Rong Hsing Research Center for Translational Medicine, National Chung Hsing University, Taipei, Taiwan; 12 Ph.D. Program in Translational Medicine, National Chung Hsing University, Taichung, Taiwan; 13 Institute of Biomedical Sciences, National Chung Hsing University, Taichung, Taiwan; 14 Department of Internal Medicine, Far Eastern Memorial Hospital, New Taipei City, Taiwan; 15 Graduate Institute of Clinical Medicine, National Taiwan University College of Medicine, Taipei, Taiwan; 16 Genomics Research Center, Academia Sinica, Taipei, Taiwan

**Keywords:** Hepatocytes, Hepatitis C

## Abstract

Data regarding the efficacy and tolerability of elbasvir/grazoprevir (EBR/GZR) for East-Asian hepatitis C virus genotype 1b (HCV GT1b) patients receiving hemodialysis were limited. We prospectively recruited 40 HCV GT1b hemodialysis patients who received EBR/GZR for 12 weeks at 6 academic centers in Taiwan. The efficacy endpoints were sustained virologic response 12 weeks off-therapy (SVR_12_) by intention-to-treat (ITT) modified ITT (mITT) analyses. Patients’ baseline characteristics, early viral kinetics and HCV resistance-associated substitutions (RASs) at HCV non-structural 3 and 5 A (NS3 and NS5A) regions potentially affecting SVR_12_ were analyzed. The tolerability for EBR/GZR was also assessed. The SVR_12_ rates by ITT and mITT analyses were 95% (38 of 40 patients; 95% confidence interval (CI): 83.5–98.6%) and 100% (38 of 38 patients; 95% CI: 90.8–100%), respectively. Patients’ baseline characteristics, on-treatment viral decline, and baseline HCV RASs did not affect SVR_12_. All patients tolerated treatment well. Among 5 patients who had serious adverse events (AEs) including one death due to on-treatment suicide and the other death due to off-therapy acute myocardial infarction, none of these events were judged related to EBR/GZR. The common AEs included upper respiratory tract infection (7.5%), fatigue (5.0%) and anorexia (5.0%). Nine (22.5%) and 8 (20.0%) patients had on-treatment hemoglobin levels of 9.0–10.0 g/dL and 7.0–9.0 g/dL. Three (7.5%) patients had on-treatment elevated alanine aminotransferase (ALT) quotient > 2.5, in whom one (2.5%) had EBR/GZR-induced late ALT elevation. No patients developed hyperbilirubinemia or hepatic decompensation. In conclusion, treatment with EBR/GZR is effective and well-tolerated for East-Asian HCV GT1b patients receiving hemodialysis.

## INTRODUCTION

Despite the introduction of universal precautions and antiviral therapies, chronic hepatitis C virus (HCV) infection remains a common health problem in patients receiving hemodialysis^1–4^. Because the potential nosocomial transmission in hemodialysis units, the HCV prevalence rates in patients receiving hemodialysis are generally higher than those in patients receiving peritoneal dialysis^5^. Compared to patients with HCV genotype 2 (GT2) infection or patients without viremia, patients with HCV GT1 infection have an increased risk of developing end-stage renal disease (ESRD)^6^. If HCV infection is left untreated, a higher proportion of HCV patients receiving hemodialysis may complicate with hepatic, infectious and cardiovascular-related mortality^7^. Based on the perspective of HCV micro-elimination, treating HCV in patients receiving hemodialysis by effective and safe antiviral regimens is mandatory to improve patients’ health outcome and to halt viral transmission^8^.

In contrast to interferon (IFN)-based antiviral therapies where the sustained virologic response (SVR) rates as well as the tolerability are far from satisfactory, the introduction of IFN-free direct acting antiviral agents (DAAs) has become the standard of care for HCV, particularly for patients receiving hemodialysis^9^. Among the approved DAA regimens, the optimized dose of sofosbuvir (SOF) cannot be recommended for patients receiving hemodialysis because the serum level of GS-331007, the inactive metabolite of SOF, is much higher in dialysis patients than that in non-dialysis patients, albeit a recent study of SOF in combination with velpatasvir (VEL) without dose reduction showed good efficacy and tolerability in patients receiving dialysis^10,11^. In contrast, elbasvir/grazoprevir (EBR/GZR) undergoes extensive hepatic metabolism and the preclinical data reveal that <1% of both EBR and GZR are renally excreted. Furthermore, a recent pharmacokinetic study indicated that the dose of EBR/GZR did not need to be adjusted in patients with ESRD^12^. In phase III C-SURFER trial which evaluated the performance of EBR/GZR for 12 weeks for HCV GT1 patients with chronic kidney disease (CKD) stage 4 or 5, the SVR at week 12 off-therapy (SVR_12_) rate was 99%. Among the subgroup patients receiving dialysis and patients with HCV GT1b infection, the SVR_12_ rates were 98.9% and 98.2%, respectively. Furthermore, most patients tolerated EBR/GZR well^13^.

Following the encouraging results from the phase III trial, the real-world studies from France and Japan evaluating the effectiveness of EBR/GZR for 12 weeks in HCV GT1b patients receiving hemodialysis showed that the SVR_12_ rates ranged from 95–100%^14–17^. However, these studies were retrospective in nature with sample sizes of around 20 patients with HCV GT1b infection, who are prevalent in hemodialysis units of East-Asia. We aimed to conduct a clinical trial to assess the performance of EBR/GZR for 12 weeks in HCV GT1b patients receiving hemodialysis in Taiwan.

## Materials and Methods

### Patients

Between June 2018 and April 2019, chronic HCV patients receiving hemodialysis were prospectively recruited at 6 academic centers in Taiwan. Patients were screened for eligibility if they were aged ≥20 years, had body mass index (BMI) between 18.5–35.0 kg/m^2^, had documented the presence of detectale HCV antibody (anti-HCV; Abbott HCV EIA 2.0, Abbott Laboratories, Abbott Park, Illinois, USA) for ≥6 months, had estimated glomerular filtration (eGFR) rate <15 mL/min/1.73m^2^ and received maintenance hemodialysis. Patients were excluded from screening if they had documented past or current presence of decompensated cirrhosis (Child-Pugh B or C), had a history of hepatocellular carcinoma (HCC), had a history of non-HCC malignancies (except for cutaneous melanoma) within 5 years of screening, had received organ transplantation (except for prior renal transplantation with graft failure), had prior exposure to DAAs, host-targeting agents or therapeutic vaccines for HCV, were pregnant, were unwilling to have contraception during the study period, or refused to provide written informed consent. Patients were not eligible for the study if they had serum HCV RNA level ≤1000 IU/mL (Cobas TaqMan HCV Test v2.0, Roche Diagnostics GmbH, Mannheim, Germany, lower limit of quantification [LLOQ]: 15 IU/mL), were infected with HCV non-GT1b genotypes (Abbott RealTi*me* HCV Genotype II, Abbott Laboratories, Abbott Park, Illinois, USA), were seropositive for hepatitis B virus (HBV) surface antigen (HBsAg, Abbott Architect HBsAg qualitative assay, Abbott Laboratories, Abbott Park, Illinois, USA) or human immunodeficiency virus (HIV) antibody (anti-HIV, Abbott Architect HIV Ag/Ab Combo, Abbott Laboratories, Abbott Park, Illinois, USA), were present with decompensated cirrhosis, malignancies or pregnancy^18^. Patients were also excluded if the hemoglobin level <10.0 g/dL, platelet count <70 × 10^9^ cells/L, international normalized ratio (INR) > 2.0, serum albumin level <3.0 g/dL, serum total bilirubin level >2.0 mg/dL, serum alanine aminotransferase (ALT) quotient >10 (upper limit of normal: 30 IU/L for males and 17 IU/L for females), or serum alfa-fetoprotein level (AFP) > 100 ng/mL^19^. The study was approved by the Ethics Committee of each participating center (National Taiwan University Hospital Ethics Committee, China Medical University & Hospital Research Ethics Center, Taipei Medical University Joint Institutional Review Board, Taichung Veterans General Hospital Institutional Review Board, and Far Eastern Memorial Hospital Research Ethics Review Committee), conducted in accordance with the principles of Declaration of Helsinki and the International Conference on Harmonization for Good Clinical Practice, and registered in ClinicalTrials.gov (NCT03420300). All patients provided written informed consent before enrollment.

### Study design

This was a one-arm, open-label, multicenter study. Data for demographics, hemogram, INR, serum albumin, total bilirubin, ALT, creatinine, AFP, anti-HCV, anti-HIV, HBsAg, HCV RNA, HCV genotype, interleukin-28B (IL28B) rs12979860 genotype (Applied Biosystems, Life Technologies Corporation, Grand Island, NY, USA), abdominal ultrasonography, and liver stiffness measurement (LSM, FibroScan^®^, Echosens, Paris, France) were collected for all screened patients^20^. The stage of hepatic fibrosis by METAVIR score was determined by LSM (F0–1: <7.0 kPa; F2: 7.0–9.4 kPa; F3: 9.5–12.4 kPa; F4: ≥ 12.5 kPa). Baseline HCV resistance-associated substitutions (RASs) for elbasvir and grazoprevir at the NS5A and NS3 regions for HCV GT1b were analyzed by population sequencing with a cut-off level of 15%^21^. If patients had on-treatment or off-therapy virologic failure, including non-response, viral breakthrough or relapse, the RAS testing was performed at the time of treatment failure. The potential drug-drug interaction (DDI) between EBR/GZR and concomitant medications was checked by HEP Drug Interaction Checker as proposed by the University of Liverpool (Liverpool, UK)^22^. If patients had comedications that were contraindicated for concomitant use, a switch to non-contraindicated comedications was performed before EBR/GZR treatment.

Patients who were eligible for the study received EBR/GZR (Zepatier^®^, 50 mg/100 mg fixed dose combination (FDC) table, Merck Sharp & Dohme (MSD) International GmbH, Ballydine, Clonmel, Ireland) 1 table daily with or without food for 12 weeks. Treatment was permanently discontinued if the patient had on-treatment viral breakthrough which was defined as patients with on-treatment undetectable viremia, but the viral load rebounded to detectable levels by continuous treatment, or had non-response which was defined as patients with persistently detectable HCV RNA beyond week 8 of treatment.

Patients received outpatient visits at treatment weeks 1, 2, 4, 6, 8, and 12, and at off-therapy weeks 4, 8, 12 to assess efficacy and tolerability. Hemoglobin, platelet count, serum total bilirubin, ALT and HCV RNA were checked at each visit. Furthermore, INR, serum albumin, eGFR, AFP, and abdominal ultrasonography were checked at on-treatment week 12 (end-of-treatment, EOT) and at off-therapy week 12.

### Efficacy

The primary efficacy endpoint was SVR_12_ by intention-to-treat (ITT) analysis, which was defined as HCV RNA level <LLOQ at off-therapy week 12, for patients who received at least one dose of EBR/GZR. The secondary efficacy endpoint was SVR_12_ by modified ITT (mITT) analysis, which excluded patients who failed to achieve SVR_12_ due to non-virologic reasons. In patients who permanently discontinued treatment, the serum HCV RNA level at the last on-treatment visit was taken as EOT viral response. Patients were considered failure to achieve SVR_12_ if they had on-treatment viral breakthrough/non-response or off-therapy viral relapse, or had missing SVR_12_.

### Tolerability

All patients received tolerability assessment for the severity and the causality of constitutional adverse events (AEs), serious AEs and laboratory abnormalities at each outpatient visit by pre-specified checklists. The severity of AEs was graded according to Common Terminology Criteria for Adverse Events (CTCAE) version 4.0. The investigators can temporarily or permanently discontinue EBR/GZR based on patients’ safety concerns. If the patient had ALT quotient ≥2.5, the alternative etiology for ALT elevation (including drug-induced hepatitis, HBV reactivation, biliary obstruction, etc.) can be assessed at investigators’ discretion. If the patient had ALT quotient ≥5, the investigators must evaluate the alternative etiology for ALT elevation. When patients presented with on-treatment ALT quotient ≥20, ALT quotient ≥10 combined with total bilirubin >3.0 mg/dL, or clinical symptoms or signs of hepatic decompensation, the EBR/GZR permanently discontinued. Dose titration for EBR/GZR was not permitted. For patients who permanently discontinued treatment, the safety summary was assessed from the beginning of treatment to the last visit. Furthermore, the common AE rates of ≥5% were also assessed. Oral nucleot(s)ides agents, such as entecavir or tenofovir, were initiated if patients were diagnosed HBV-associated hepatitis flare, defined as the combined presence of HBV reactivation (HBsAg seropositivity or HBV DNA level ≥100 IU/mL) and ALT quotient ≥5^23^.

### Drug compliance

The adherence of EBR/GZR was assessed by self-reporting diaries and pill counts, which were calculated as the number of pills taken (the number of pills dispensed minus the number of pills counted) at each outpatient visit. The compliance was presented as the total pills consumed during the study period divided by the scheduled 84 pills.

### Statistical Analyses

Statistical Program for Social Sciences (SPSS Statistics Version 23.0, IBM Corp., Armonk, New York, USA) was used for statistical analyses. We assumed a total of 40 patients would provide an SVR rate in HCV GT1b patients who receive EBR/GZR for 12 weeks to be 95% with a 95% lower confidence bound to be 83%, and with a 10% of response rate higher than the 95% upper confidence bound to be 73% in the historical SVR rate of 64% in East-Asian HCV GT1b patients treated by pegylated IFN plus ribavirin (RBV)^24^. The baseline patient characteristics were shown in median (range) and percentages when appropriate. The on-treatment and off-therapy viral response rates were shown in number and percentages with 95% confidence interval (CI). Patients’ baseline characteristics, early viral kinetics and HCV RASs potentially affecting SVR_12_ were analyzed. The safety summaries were shown in number and percentages when appropriate.

## Results

### Patient characteristics

**Figure 1:**
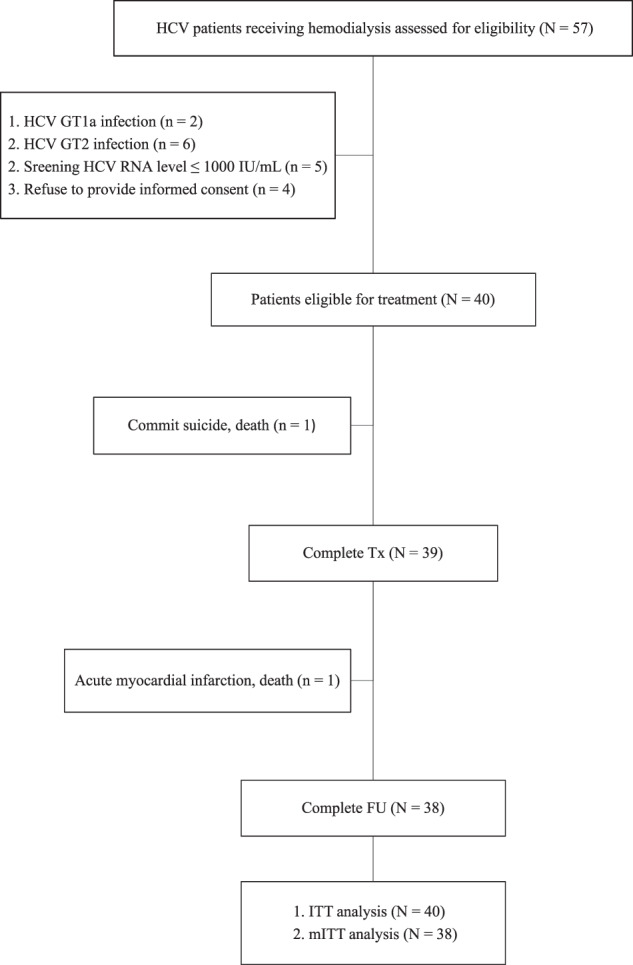
Study flow.

Of the 57 patients assessed for eligibility, 17 were excluded from the study because of HCV non-GT1b infection (n = 8; 2 with HCV GT1a and 6 with HCV GT2 infections), serum HCV RNA level ≤1000 IU/mL (n = 5), and refusal for providing informed consent (n = 4). The remaining 40 patients were eligible for the study (Fig. 1). Table 1 shows the baseline characteristics. The median age was 64 years and 23 (58%) patients were males. Thirty-five (88%), 5 (13%), and 6 (15%) patients were treatment-naïve, had a prior history of renal transplantation, and had IL28B rs12979860 non-CC genotypes. The median serum total bilirubin level and ALT quotient were 0.5 mg/dL and 0.7, respectively. The median log_10_ HCV RNA level was 5.60, and 11 (27%) patients had baseline HCV RNA levels of >800,000 IU/mL. Twenty (50%), 14 (35%), 2 (5%), and 4 (10%) patients had a fibrosis stage of F0–1, F2, F3, and F4, respectively. Thirty-three (83%) and 7 (18%) had baseline HCV RASs at NS3 and NS5A regions, respectively. Among patients with baseline RASs, all had single amino acid substitution at any specific locus at NS3 or NS5A region (Supplementary Table 1). Furthermore, all patients with RASs showed curve patterns on electropherograms as mixed wild/mutant curves at NS3 or NS5A region.

**Table 1:** Baseline Patient Characteristics.

Characteristics†	Patient (N = 40)
Age, year, median (range)	64 (32–85)
Male	23 (58)
Treatment-naïve	35 (88)
IFN-based treatment-experienced	5 (12)
History of renal transplantation	5 (13)
Diabetes mellitus	13 (33)
Hypertension	23 (58)
Dyslipidemia	8 (20)
Hemoglobin, g/dL, median (range)	11.8 (10.1–14.8)
Absolute neutrophil count, 10^9^ cells/L, median (range)	3.7 (2.0–7.1)
Platelet count, 10^9^ cells/L, median (range)	196 (100–345)
INR, median (range)	1.01 (0.89–1.26)
Albumin, g/dL, median (range)	4.3 (3.4–5.1)
Total bilirubin, mg/dL, median (range)	0.5 (0.1–1.0)
ALT quotient, median (range)	0.7 (0.2–3.9)
Creatinine, mg/dL, median (range)	7.3 (4.2–15.4)
eGFR, mL/min/1.73m^2^, median (range)	7 (3–10)
IL28B rs12979860 non-CC genotypes	6 (15)
HCV RNA, log_10_ IU/mL, median (range)	5.60 (4.18–7.28)
HCV RNA > 800,000 IU/mL	11 (27)
Liver stiffness measurement (FibroScan), kPa, median (range)	7.1 (3.3–57.9)
**Fibrosis stage (by liver stiffness measurement)**	
F0–1	20 (50)
F2	14 (35)
F3	2 (5)
F4	4 (10)
**Resistance-associated substitution**	
NS3	33 (82)
NS5A	7 (18)

IFN: interferon; INR: international normalized ratio; ALT: alanine aminotransferase; eGFR: estimated glomerular filtration rate; IL: interleukin; HCV: hepatitis C virus; RNA: ribonucleic acid.

^†^Values are numbers (percentages) unless otherwise indicated.

### Efficacy

At on-treatment weeks 4, 39 patients (97.5%; 95% CI: 87.1–99.6%) had serum HCV RNA levels <LLOQ. The serum HCV RNA levels were all <LLOQ beyond on-treatment weeks 6. The SVR_12_ rates by ITT and mITT analyses were 95.0% (38 of 40 patients; 95% CI: 83.5–98.6%) and 100% (38 of 38 patients; 95% CI: 90.8–100%), respectively (Table 2). The baseline patient characteristics, on-treatment viral decline, or baseline HCV RASs at NS3 or NS5A region did not affect the SVR_12_ rates.

**Table 2:** On-treatment and Off-therapy Virologic Responses.

HCV RNA level < LLOQ	Patient	
	n/N (%)	95% CI
**During treatment**		
Week 1	22/40 (55.0)	39.8–69.3
Week 2	35/40 (87.5)	73.9–94.5
Week 4	39/40 (97.5)	87.1–99.6
Week 6	40/40 (100)	91.2–100
Week 8	40/40 (100)	91.2–100
Week 12^†^	39/40 (97.5)	87.1–99.6
EOT^†^	40/40 (100)	91.2–100
**After treatment**		
SVR_4_^‡^	38/38 (100)	90.8–100
SVR_8_^‡^	37/37 (100)	90.6–100
SVR_12_ (ITT)	38/40 (95.0)	83.5–98.6
SVR_12_ (mITT)	38/38 (100)	90.8–100
**Reason for non-SVR**_**12**_, **n**		
Lost to follow-up or discontinued early due to reasons other than virologic failure	2	

LLOQ: lower limit of quantification; EOT: end-of-treatment; CI: confidence interval.

^†^One patient committed suicide at on-treatment week 10. The serum HCV RNA level was undetectable at the last visit (on-treatment week 8).

^‡^One patient died of suicide at on-treatment week 10 and did not have off-therapy HCV RNA data. One died of acute myocardial infarction at off-therapy week 8 and did not have SVR_8_ and SVR_12_ HCV RNA data. One patient was lost to follow-up during off-therapy weeks 4 to 8 and did not have SVR_4_ and SVR_8_ HCV RNA data. The patient resumed follow-up at off-therapy week 12.

### Tolerability

Table 3 shows the safety summary. All patients completed EBR/GZR treatment for 12 weeks except one who died of suicide at on-treatment week 10 after losing vision from retinal hemorrhage at on-treatment week 8. One patient who completed treatment but died of acute myocardial infarction at off-therapy week 8 (Fig. 1). Another 3 patients developed serious AEs which did not lead to patients’ deaths and were judged not related to EBR/GZR, including one non-cirrhotic patient admitted due to cardiac arrhythmia (atrial fibrillation) at on-treatment week 4, and the other two cirrhotic patients admitted due to non-variceal gastrointestinal bleeding at off-therapy week 4, and hepatocellular carcinoma at off-therapy week 4. The common AEs included upper respiratory tract infection (7.5%), fatigue (5.0%) and anorexia (5.0%). All the common AEs were mild in grade. Nine (22.5%) and eight (20.0%) patients had on-treatment hemoglobin levels of 9.0–10.0 g/dL and 7.0–9.0 g/dL. None had on-treatment total bilirubin level >1.5 mg/dL. One patient with ALT quotient between 10.0–15.0 at on-treatment week 8, which was related to EBR/GZR-induced ALT elevation after etiology assessment. The other 2 patients with ALT quotient between 2.5–5.0 at on-treatment weeks 2 and 4, which were judged not related to EBR/GZR or to HBV reactivation after etiology assessments. All the 3 patients were asymptomatic and did not have total bilirubin levels >3.0 mg/dL or symptoms/signs of hepatic decompensation. Furthermore, the ALT levels normalized 2 weeks later in all without stopping EBR/GZR.

**Table 3:** Safety Summary.

Variable, n (%)	Patient (N = 40)
**Permanent treatment discontinuation**	1 (2.5)
**Serious adverse event**^†^	5 (12.5)
**DAA-related serious adverse event**	0 (0)
**Death**^†^	2 (5.0)
**DAA-related death**	0 (0)
**Adverse event in ≥ 5% of patients**	
Upper respiratory tract infection	3 (7.5)
Fatigue	2 (5.0)
Anorexia	2 (5.0)
**Laboratory abnormalities**	
Hemoglobin	
9.0–10.0 g/dL	9 (22.5)
7.0–9.0 g/dL	8 (20.0)
**Total bilirubin**	
>1.5 mg/dL	0 (0)
**ALT quotient**^‡^	
2.5–5.0	2 (5.0)
5.0–10.0	0 (0)
10.0–15.0	1 (2.5)

^†^Suicide (n = 1), acute myocardial infarction (n = 1), cardiac arrhythmia (n = 1), non-variceal gastrointestinal bleeding (n = 1), and hepatocellular carcinoma (n = 1), The first two serious adverse events resulted in patients’ deaths.

^‡^One patient with ALT quotient between 10.0–15.0 at on-treatment week 8, which was considered probably related to EBR/GZR. The other 2 patients with ALT quotients between 2.5–5.0 at on-treatment weeks 2 and 4, which were considered not related to EBR/GZR. All patients were asymptomatic and did not have elevated total bilirubin levels. All patients continued EBR/GZR and all the ALT elevations resolved 2 weeks later.

### Drug compliance

Regarding drug compliance, 33 (83.5%), 5 (12.5%), 1 (2.5%) and 1 (2.5%) patients had consumed 100%, 95–99.9%, 90–94.9%, and 85–89.9% of the scheduled pills.

## Discussion

In contrast to SOF-based DAAs which undergo extensive renal excretion, the combination of NS3 protease inhibitor (PI) with NS5A inhibitor is appealing to practitioners in the care of HCV among patients receiving hemodialysis because both agents are mainly metabolized by the liver^11,12^. Our study demonstrated that the SVR_12_ rate of EBR/GZR for 12 weeks was excellent (95%) for East-Asian HCV GT1b patients receiving hemodialysis by ITT analysis. Furthermore, by excluding 2 patients who died of non-virologic reasons, all the remaining 38 patients achieved SVR_12_. Our efficacy endpoints were in line with prior reports from global clinical trials and real-world studies from France and Japan, implying race may not play a role on the treatment responses^13–17^. The lower 95% confidence bound of SVR_12_ rate in our study was 83.5%, which was 19.5% higher than the SVR rate of 64% by pegylated IFN plus RBV therapy in East-Asian HCV GT1b patients receiving hemodialysis. In contrast to the high proportions of treatment-emergent AEs by IFN-based therapies, the tolerability of EBR/GZR in our study was excellent. Although the phase III C-SURFER trial recruited a sizable number of patients receiving EBR/GZR, only 5 (4.5%) patients in the immediate treatment group were Asians. Furthermore, the real-world studies for Asian hemodialysis HCV GT1b patients receiving EBR/GZR were all reported from Japan with retrospective design and small sample size. In contrast, our patients were prospectively selected by well-defined inclusion and exclusion criteria, and all study procedures were assessed by pre-specified protocol, which can provide an accurate and unbiased clinical assessment of EBR/GZR in this special clinical setting. Taken together, EBR/GZR can be a preferred choice for treating East-Asian HCV GT1b patients receiving hemodialysis in terms of efficacy and tolerability.

Our study recruited patients with baseline HCV RNA of >800,000 IU/mL (27%), IL28B non-CC genotypes (15%), and baseline HCV RASs at NS3 (82%) and NS5A regions (18%), which might compromise the SVR_12_ rate^25^. However, none had on-treatment or off-therapy virologic failures by EBR/GZR, implying that the baseline patient and viral factors did not affect the treatment response. Although we adopted population sequencing with a cut-off level of 15% to detect RAS from electropherograms instead of using next-generation sequencing (NGS) to precisely determine the proportions of RASs in NS3 and NS5A loci, our results indicated that pre-treatment RAS testing may not be needed for HCV GT1b patients receiving hemodialysis before EBR/GZR treatment^13^. In addition, 97.5% of our patients had HCV RNA levels <LLOQ at on-treatment week 4, and therefore the on-treatment HCV viral kinetics seemed not to have a role in predicting SVR_12_^13,16^.

Regarding tolerability, all patients completed the assigned treatment except one who died of suicide at on-treatment week 10. Although serious AEs occurred in 5 (12.5%) patients including two deaths (suicide during therapy and acute myocardial infarction during off-therapy follow-up), none were judged related to EBR/GZR. With regard to laboratory abnormalities, our data were in line with prior reports that about 15–40% of these patients had on-treatment hemoglobin levels <10 g/dL, which were attributed to renal anemia^13,14,16^. In contrast to patients treated by paritaprevir/ritonavir/ombitasvir plus dasabuvir (PrOD) which may result in higher risks of on-treatment hyperbilirubinemia, none in our study had ≥ grade 2 on-treatment hyperbilirubinemia, suggesting GZR did not significantly inhibit organic anion transport proteins (OATPs)^26,27^. One patient (2.5%) in our study presented with EBR/GZR-induced late ALT elevation at on-treatment week 8, which was in line with an integrated analysis of phase 2 and 3 trials showing the proportion of EBR/GZR-induced late ALT elevation to be 0.8% and 2.4% in overall and Asian populations, respectively^28^. However, this patient was asymptomatic without signs of hepatic decompensation, and the ALT elevation resolved with continuous use of EBR/GZR. With regard to the assessment of HBV reactivation, we did not routinely check serum HBV DNA level at each outpatient visit to early detect occult HBV infection because the risks of HBV reactivation or HBV-associated hepatitis in these patients were only 1.4% and 0.5%, respectively^29^. Taken together, the tolerability was excellent for hemodialysis patients receiving EBR/GZR for HCV.

In conclusion, our multicenter prospective study confirms that EBR/GZR for 12 weeks is effective for East-Asian HCV GT1b patients receiving hemodialysis. Furthermore, the tolerability is also excellent for these patients receiving EBR/GZR.

## Supplementary information

Supplementary Table 1.
