# Supplementary material for: Elbasvir/grazoprevir for hepatitis C virus genotype 1b East-Asian patients receiving hemodialysis
Source: Sci Rep. 2020 Jun 8;10:9180. doi: 10.1038/s41598-020-66182-8 (PMC7280513; doi:10.1038/s41598-020-66182-8)
Supplement: Supplementary file 1 — Supplementary Table 1. [file 41598_2020_66182_MOESM1_ESM.docx]

**Elbasvir/grazoprevir for hepatitis C virus genotype 1b East-Asian patients receiving hemodialysis**

Chen-Hua Liu, Cheng-Yuan Peng, Yu-Jen Fang, Wei-Yu Kao, Sheng-Shun Yang, Cheng-Kuan Lin, Hsueh-Chou Lai, Wen-Pang Su, Sheng-Uei Fang, Chun-Chao Chang, Tung-Hung Su, Chun-Jen Liu, Pei-Jer Chen, Ding-Shinn Chen, Jia-Horng Kao

**Supplementary Table 1. List of RASs in NS3 and NS5A amino acid positions**

| **RAS region** | **Amino acid substitution†** | **Patients with RAS, n/N (%)** |
| --- | --- | --- |
| **NS3** | V36A/I/L/M | 0//40 (0) |
|  | Q41L/R | 0/40 (0) |
|  | F43/S | 1/40 (3) |
|  | T54A/C/G/S | 0/40 (0) |
|  | V55A/I | 1/40 (3) |
|  | Y56F | 27/40 (68) |
|  | Q80L/R | 11/40 (28) |
|  | V107I | 0/40 (0) |
|  | S122A/G/R | 8/40 (20) |
|  | R155E/K/N/Q/S | 1/40 (3) |
|  | A156G/S | 0/40 (0) |
|  | D168E/N/S | 0/40 (0) |
|  | V170A/I/T | 12/40 (30) |
| **NS5A** | L28A/G/M/T | 1/40 (3) |
|  | Q30H/K/R/Y | 2/40 (5) |
|  | L31F/I/M/V | 1/40 (3) |
|  | P32del | 0/40 (0) |
|  | H58D | 2/40 (5) |
|  | A92K | 1/40 (3) |
|  | Y93H/N/S | 1/40 (3) |

† The potential amino acid substitutions were shown at NS3 and NS5A loci. Patients with multiple amino acid substitutions at any NS3 or NS5A locus were shown in independent footnotes.
